# Supplementary material for: Physiotherapist’ job performance, impression management and organizational citizenship behaviors: An analysis of hierarchical linear modeling
Source: PLoS One. 2021 May 21;16(5):e0251843. doi: 10.1371/journal.pone.0251843 (PMC8139475; doi:10.1371/journal.pone.0251843)
Supplement: S1 Table — (DOCX) [file pone.0251843.s001.docx]

S1 Table. This is the S1 Table Descriptive statistics, correlation coefficients, and reliability coefficient of the research variables. This is the S1 Table legend.

**Table 1. Descriptive statistics, correlation coefficients, and reliability coefficient of the research variables**

| Research variables | M | SD | Cronbach^，^α | Research variables’ related data | | |
| --- | --- | --- | --- | --- | --- | --- |
|  |  |  |  | (1) | (2) | (3) |
| Impression management | 2.84 | 0.61 | 0.93 | 1 |  |  |
| Organizational citizenship behaviors | 3.29 | 0.35 | 0.75 | 0.417^***^ | 1 |  |
| Job performance | 3.80 | 0.46 | 0.95 | 0.204^***^ | 0.679^***^ | 1 |

Note: ^＊^*p*＜.05, ^＊＊^*p*＜.01, and^＊＊＊^*p*＜.001.
