# Supplementary material for: Physiotherapist’ job performance, impression management and organizational citizenship behaviors: An analysis of hierarchical linear modeling
Source: PLoS One. 2021 May 21;16(5):e0251843. doi: 10.1371/journal.pone.0251843 (PMC8139475; doi:10.1371/journal.pone.0251843)
Supplement: S2 Table — (DOCX) [file pone.0251843.s002.docx]

S2 Table. This is the S1 Table Description of the sample (n = 523).

This is the S2 Table legend.

**Table 2. Description of the sample (n = 523)**

|  | Category | Participants | ％ |
| --- | --- | --- | --- |
| Physiotherapist |  | 523 | 100 |
| SEX | Male | 215 | 41.1 |
|  | Female | 308 | 58.9 |
| Office unit | Medical center | 43 | 8.22 |
|  | Regional hospital | 73 | 13.96 |
|  | Local education | 130 | 24.86 |
|  | Clinic | 223 | 42.64 |
|  | Others | 54 | 10.32 |
