# Supplementary material for: Physiotherapist’ job performance, impression management and organizational citizenship behaviors: An analysis of hierarchical linear modeling
Source: PLoS One. 2021 May 21;16(5):e0251843. doi: 10.1371/journal.pone.0251843 (PMC8139475; doi:10.1371/journal.pone.0251843)
Supplement: S3 Table — (DOCX) [file pone.0251843.s003.docx]

S3 Table. This is the S3 Table 3. Regression analysis of impression management and organizational citizenship behaviors. This is the S3 Table legend.

**Table 3. Regression analysis of impression management and organizational citizenship behaviors**

| Criterion  Predictor | Organizational citizenship behaviors |
| --- | --- |
| Ingratiation | .103^***^ |
| Opinion conformity | −.013^***^ |
| Rendering Favors | .058^***^ |
| Self-presentation | .346^***^ |
| F | 32.318^***^ |
| R^2^ | .194 |

^＊＊＊^*p*＜.001.
