# Supplementary material for: Physiotherapist’ job performance, impression management and organizational citizenship behaviors: An analysis of hierarchical linear modeling
Source: PLoS One. 2021 May 21;16(5):e0251843. doi: 10.1371/journal.pone.0251843 (PMC8139475; doi:10.1371/journal.pone.0251843)
Supplement: S4 Table — (DOCX) [file pone.0251843.s004.docx]

S4 Table. This is the S4 Table 4. Regression analysis of organizational citizenship behaviors and job performance. This is the S4 Table legend.

**Table 4. Regression analysis of organizational citizenship behaviors and job performance**

| Criterion  Predictor | Job performance |
| --- | --- |
| Conscientiousness | .149^***^ |
| Sportsmanship | −.301 |
| Civic virtue | .121^***^ |
| Courtesy | .299^***^ |
| Altruism | .369^***^ |
| F | 157.085^***^ |
| R^2^ | .603 |

Note:^＊^*p*＜.05, ^＊＊^*p*＜.01, and ^＊＊＊^*p*＜.001.
