# Supplementary material for: Physiotherapist’ job performance, impression management and organizational citizenship behaviors: An analysis of hierarchical linear modeling
Source: PLoS One. 2021 May 21;16(5):e0251843. doi: 10.1371/journal.pone.0251843 (PMC8139475; doi:10.1371/journal.pone.0251843)
Supplement: S5 Table — (DOCX) [file pone.0251843.s005.docx]

S5 Table. This is the S5 Table 5. Regression analysis of impression management and organizational citizenship behaviors on job performance.

This is the S5 Table legend.

**Table 5. Regression analysis of impression management and organizational citizenship behaviors on job performance**

| Invest in the variable | Model 1(β) | Model 2(β) |
| --- | --- | --- |
| Ingratiation | .230^***^ | .163^***^ |
| Opinion conformity | −.324^***^ | −.104^*^ |
| Rendering Favors | .101^**^ | .005 |
| Self-presentation | .210^***^ | −.098 |
| Conscientiousness |  | .163^***^ |
| Sportsmanship |  | −.016 |
| Civic virtue |  | .127^***^ |
| Courtesy |  | .268^***^ |
| Altruism |  | .398^***^ |
| F | 15.198^***^ | 92.910^***^ |
| △F | 15.198^***^ | 138.896^***^ |
| R^2^ | .105 | .620 |
| △R^2^ | .098 | .613 |

Note: ^＊^*p*＜.05, ^＊＊^*p*＜.01, and ^＊＊＊^*p*＜.001.
