# Supplementary material for: Physiotherapist’ job performance, impression management and organizational citizenship behaviors: An analysis of hierarchical linear modeling
Source: PLoS One. 2021 May 21;16(5):e0251843. doi: 10.1371/journal.pone.0251843 (PMC8139475; doi:10.1371/journal.pone.0251843)
Supplement: S6 Table — (DOCX) [file pone.0251843.s006.docx]

S6 Table. This is the S6 Table 6. Hierarchical linear model of the various indicators.

This is the S6 Table legend.

**Table 6. Hierarchical linear model of the various indicators**

|  | γ00 | γ01 | γ10 | γ20 | τ00 | τ11 | σ^2^ | deviance |
| --- | --- | --- | --- | --- | --- | --- | --- | --- |
| 1. Null model  L1: Y(JP) = β0j + rij  L2: β0j = γ00 + U0j | 3.79^***^  (0.021) |  |  |  | 0.027^***^ |  | 0.215 | 686.222 |
| 2. Intercepts-as-outcomes  models  (1) AIM－JP  L1: Y(JP) = β0j + rij  L2: β0j = γ00 + γ01(AIM) + U0j | 3.76^***^  (0.29) | 0.172^*^  (0.09) |  |  | 0.013^*^ |  | 0.21 | 684.708 |
| (2)AIM－OCB－JP  L1: Y(JP) = β0j + β1(OCB) + rij  L2: β0j = γ00 + γ01(AIM) + U0j  β1i = γ10 + U1i | 0.74^***^  (0.173) | 0.037^*^  (0.068) | 0.912^***^ (0.051) |  | 0.11^*^ | 0.09^*^ | 0.11 | 337.839 |
| 3. Slopes-as-outcomes  model  (1) AIM－OCB－JP  L1: Y(JP) = β0j + β1(OCB) + β2 (IM) + rij  L2: β0j = γ00 + γ01 (AIM) + U0j  β1i = γ10 + γ11 (AIM) + U1i | 0.816^***^  (0.18) | 0.11^*^  (0.08) | 0.08^**^  (0.027) | 0.96^***^  (0.05) | 0.09^*^ | 0.02 | 0.1 | 329.604 |

Note：L1 = Individual-level；L2 = Overall level；IM = impression management；AIM = aggregate impression management；OCB = organizational citizenship behaviors；JP = job performance

Note: ^＊^*p*＜.05, ^＊＊^*p*＜.01, and ^＊＊＊^*p*＜.001.
