# Supplementary material for: Physiotherapist’ job performance, impression management and organizational citizenship behaviors: An analysis of hierarchical linear modeling
Source: PLoS One. 2021 May 21;16(5):e0251843. doi: 10.1371/journal.pone.0251843 (PMC8139475; doi:10.1371/journal.pone.0251843)
Supplement: Attachment — Submitted filename: Reviewer20201222.docx [file pone.0251843.s008.docx]

PLOS ONE

Manuscript Number: PONE-D-20-16522
Manuscript Title: Physiotherapist’ Job Performance, Impression Management and Organizational Citizenship Behaviors: An Analysis of Hierarchical Linear Modeling

**Authors’ Response to Editor**

We have carefully considered the concerns and questions about research design, research methods, and other issues in our manuscript and followed your instructions to further revise the paper. We hope that these concerns have been adequately addressed. Please find our itemized responses to reviewers‟ comments below. Again, many thanks for your kind guidance for making the revision. We have enjoyed doing it. If further revision is needed, please let us know. The blue color texts in the manuscript are revisions according to reviewer.

**Authors’ Response to Reviewer 1**

We thank you for your constructive and detailed comments on how to improve our paper. We have followed your comments to revise our paper. The details are as follows (one-by-one, we put your specific comments in bold, followed by our response):

● **One of my main concerns is a possible common method bias. Because physiotherapists completed all the measures, how could the authors be sure that common method bias was avoided?**

**[Authors’ Response]**

Thanks for raising this issue. we have revised in page 7. Line 124-132.

.…This research ensured avoiding the problems of homologous bias and common method variation in the collection of data. It is considered that the collection and measurement of performance indicators should be recommended after the high-performance work system [32]. The questionnaires used in this study were distributed in two stages. The first stage was to measure the variables of the high-performance work system. The second questionnaire was executed two months after the first was issued. Since this research adopted a cross-layer analysis, the unit of the second questionnaire was primarily the unit that was issued the first time and the professional performance was carried out..….

32.	Wright PM, Gardner TM, Moynihan LM, Allen MR. The relationship between HR practices and firms performance: Examining causal order. Personnel Psychology. 2005;58(2): 409–446.

● **In the abstract the authors state that "At the group level, impression management had a conditioning effect on organizational citizenship behaviors and job performance." Please clarify in what way.**

**[Authors’ Response]**

Thanks for raising this important question. Please see the abstract in page 1. Line 17-20.

….. In terms of statistical methods, group-level variables act as moderators, which affects the power of individual-level explanatory variables on outcome variables, i.e., the influence of the slope. The job behaviors of physiotherapists entail direct service and their performance is closely related to organizational development..…..

And page19. Line 247-251.

…. There is an important assumption in the multi-level model analysis that may affect the interpretation of individual-level variables for outcome variables, i.e., multi-level models have cross-level interactions [38]. In terms of statistical methods, group-level variables act as moderators, which affects the power of individual-level explanatory variables on outcome variables, i.e., the influence of the slope [39]..….

38.	Haw Jeng C. Multilevel latent variable modeling of contextual variables: Application of multilevel structural equation modeling on rater effects. Chinese Journal of Psychology. 2007;49(4): 383–405. doi:10.6129/CJP.2007.4904.05

39.	Raudenbush SW, Bryk AS. Hierarchical linear models: Applications and data analysis methods, 2nd ed. Newbury Park, CA: Sage. 2002.

●**In the conclusion to the abstract the authors state that "The job behaviors of physiotherapists entail direct service and their performance is closely related to organizational development."- This sentence is vague and I do not understand the connection between physiotherapists and organizational development. What does it mean that their behavior is closely related to organizational development? In what way? How does physiotherapists' job differ from any other job with respect to organizational development?**

**[Authors’ Response]**

Thanks for raising this important question. Please see the abstract in page 2. Line 21-24.

….. Impression management gives people certain purposes and behaviors while organizational citizenship behaviors are a type of non-self-seeking, selfless dedication behaviors. Therefore, the motivation of physiotherapists who demonstrate organizational citizenship behaviors should be further explored.….

●**The authors should provide more references supporting their claims. For example, even in the beginning of the introduction (p. 5 lines 66-69): "Current medical environments are patient-centered, and rehabilitation medical services and needs have increased significantly (Author? Date ?). Moreover, in an era of comprehensive competition, medical institutions’ quality is an important strategic indicator of competitiveness in the medical industry." (Author? Date?**

**[Authors’ Response]**

Thanks for pointing out the limitation of our review work. We have now updated the literature review with more recent published works. Please see page 2. Line31-34.

Current medical environments are patient-centered, and rehabilitation medical services and needs have significantly increased [1, 2]. Moreover, in an era of comprehensive competition, the quality of medical institutions is an important strategic indicator of competitiveness in the industry [3].

Below is a list of paper we added to the literature review section:

1.	Bates DW, Bitton A. The future of health information technology in the patient-centered medical home. Health affairs. 2010;29(4): 614–621.

2.	Zhang H, Shen Q. The current situation of rehabilitation medical service system in China: Problems and challenges. Business and management research. 2014;3(2): 42–46.

3. Rivers PA, Glover SH. Health care competition, strategic mission, and patient satisfaction: research model and propositions. Journal of health organization and management. 2008.

●**The authors state on p. 5 lines 69-71: "In terms of medical management, factors affecting physiotherapists’ attitude should be completely understood to improve service quality." – how can attitudes be "completely understood" – I suggest using more accurate terms throughout the paper. The rationale for the study and the hypotheses should be better developed and presented in a more integrative way.**

**[Authors’ Response]** Thanks for your good suggest. we have revised in page 2. Line34-36.

In medical management, it is necessary to comprehensively understand the factors that affect the work attitude of physiotherapists to improve the quality of services.

●**On p. 6 lines 86-88 the authors state that "They [OCB] are spontaneous behaviors that are not described in job manuals and occur when employees assist each other to accomplish their tasks." – I do not agree that organizational citizenship behavior is only "spontaneous behaviors". Please provide references from the literature on OCB, and in particular supporting this notion of 'spontaneous behaviors' and expand the definition.**

**[Authors’ Response]**

Thanks for raising this significant issue. Please see page 4. Line 51-56. Based on the literature review, the text is revised as follows:

… Mcshane and Glinow point out that these behaviors include active participating in organizational activities, selflessly assisting colleagues, and reducing unnecessary conflicts [4]. Konovsky and Pugh opine that the performance of organizational citizenship behaviors is not subject to the requirements of the employee’s task and the ability to judge their performance; they also claim that employees do not mind whether they will be rewarded [18].….

18.	Konovsky MA, Pugh SD. Citizenship behavior and social exchange. Acad Manage J.1994; 37(3), 656–669.

●**The authors cite references from books (not updated) on organizational behavior (i.e. No. 19) – "****Robbins and Judge (2011)." – The authors should conduct a better and more updated literature search to support their arguments.**

**[Authors’ Response]**

Thanks for pointing out our question. We have now updated the literature review with more recent published works. Please see page 4. Line 64-67.

… Robbins and Judge [23,24] view the impression management strategy as the displayed behavior before making a request. In other words, it refers to the way one treats work performance as a professional and how actively they participate in their work, and what they think is important to them in their work.….

24.	Robbins SP, Judge TA. Organizational behavior*.* 18th ed. New Jersey: Pearson. 2019.

●**On p. 8 lines 124-125 the authors claim that "Therefore, this study wants to understand the motivation of physiotherapist's impression management" Didn’t the authors want to examine the effects of impression management? This sentence in misleading.**

**[Authors’ Response]**

Thanks for your comments. We revised the sentence in page 5. Line 93-95.

…. Therefore, this study seeks to examine the effects of physiotherapists’ impression management, study whether organizational citizenship behavior affects their job performance, and identify the relationship between various aggregates.

…..

●**In their hypotheses the authors use the word "impact". Because they did not use an experimental design they can't deduce causality and thus should only use terms such as "associate" or "predict".**

**[Authors’ Response]**

Thanks for the recommendations for wording, which we have revised accordingly in the manuscript. Thanks. Please see page 6. line 104-114.

…. Hypothesis 1: Impression management positively associates organizational citizenship behaviors (H1);

Hypothesis 2: Organizational citizenship behaviors positively associate job performance (H2);

Hypothesis 3: Organizational citizenship has a mediating effect between impression management and job performance (H3);

Hypothesis 4: Aggregate impression management positively associates organizational citizenship behaviors (H4);

Hypothesis 5: Aggregate impression management positively associates job performance (H5); and

Hypothesis 6: Aggregate impression management has a conditioning effect on organizational citizenship and job performance (H6)..…..

●**In the method section the authors should include a specific description of each of their measures according to APA guidelines.**

**[Authors’ Response]**

We revised in page 6. Line 132-140 according to your comments.

… In the questionnaire survey, the scales for impression management, organizational citizenship behaviors, and job performance were adopted from Kumar and Beyerlein [33], Polsakoff et al. [34], and Motowidlo and Van Scotter [35], respectively. The responses are measured on a five-point Likert scale, from largely disagree to largely agree, with 1 to 5 points. The correlation coefficient between impression management and organizational citizenship behaviors was 0.417, between organizational citizenship behaviors and job performance was 0.679, and between impression management and job performance was 0.204. All values showed a significant positive correlation and reliability above 0.7 (Table 1).…..

●**The results are not presented according to the APA format. Crucially, that authors should present the exact p values. In addition, the reliabilities should come after the measures subsection (not in a Table of means, SD and inter-correlations).**

**[Authors’ Response]**

Thanks for the recommendations. We revised in page 9-10. Line 147-155, according to your comments.

…. The relevant analysis and reliability tests were conducted on the sample using the SPSS20 statistical software for Windows. The hierarchical linear modeling (HLM) was then constructed using HLM 7.01 for regression analysis.

The analysis included (1) a null model for calculating the intra-class correlation coefficient (ICC) to determine differences among the overall levels and (2) intercepts- and slope-as-outcome for exploring the impact of all independent variables on the effect of dependent variables at the individual level and using the overall level independent variables to explain the variability of dependent variable at the individual level.….

●**Again on p. 12 and in the discussion section the authors use the word "impact" although they can't deduce causality.**

**[Authors’ Response]**

Thanks for the recommendations for wording, which we have revised accordingly in the manuscript. Please see page 18. Line234-236.

… The regression analysis showed that impression management had a significant positive effect on organizational citizenship behaviors and job performance,….

●**In the discussion the authors state that "…physiotherapists had greater impression management, they were more motivated to manipulate organizational citizenship behaviors to change the perception of others toward them…" – I don’t think that the authors used any measure that can show that physiotherapists manipulated their organizational citizenship behaviors. They authors did not examine this.**

**[Authors’ Response]**

Thanks for the recommendations. We revised in page 18. Line 238-242, according to your comments.

…. The HLM analysis supported the cross-level conditioning effect of impression management, which suggests that when physiotherapists had greater impression management, they were more associates to affect organizational citizenship behaviors to change the perception of others toward them, hence increasing their chance of achieving their goals.…..

●PLOS authors have the option to publish the peer review history of their article ([what does this mean?](https://journals.plos.org/plosone/s/editorial-and-peer-review-process#loc-peer-review-history)). If published, this will include your full peer review and any attached files.

**[Authors’ Response]**

Yes: Dr. wan yun huang
